# Attentional Bias for Reward and Punishment in Overweight and Obesity: The TRAILS Study

**DOI:** 10.1371/journal.pone.0157573

**Published:** 2016-07-08

**Authors:** Nienke C. Jonker, Klaske A. Glashouwer, Brian D. Ostafin, Madelon E. van Hemel-Ruiter, Frédérique R. E. Smink, Hans W. Hoek, Peter J. de Jong

**Affiliations:** 1 Department of Clinical Psychology and Experimental Psychopathology, University of Groningen, Groningen, The Netherlands; 2 Accare, Child and Adolescent Psychiatry, Center for Eating Disorders, Accare, The Netherlands; 3 Verslavingszorg Noord-Nederland, Groningen, The Netherlands; 4 Parnassia Psychiatric Institute, The Hague, The Netherlands; 5 Department of Psychiatry, University Medical Center Groningen, University of Groningen, Groningen, The Netherlands; 6 Department of Epidemiology, Mailman School of Public Health, Columbia University, New York, New York, United States of America; Old Dominion University, UNITED STATES

## Abstract

More than 80% of obese adolescents will become obese adults, and it is therefore important to enhance insight into characteristics that underlie the development and maintenance of overweight and obesity at a young age. The current study is the first to focus on attentional biases towards rewarding and punishing cues as potentially important factors. Participants were young adolescents (*N* = 607) who were followed from the age of 13 until the age of 19, and completed a motivational game indexing the attentional bias to general cues of reward and punishment. Additionally, self-reported reward and punishment sensitivity was measured. This study showed that attentional biases to cues that signal reward or punishment and self-reported reward and punishment sensitivity were not related to body mass index or the change in body mass index over six years in adolescents. Thus, attentional bias to cues of reward and cues of punishment, and self-reported reward and punishment sensitivity, do not seem to be crucial factors in the development and maintenance of overweight and obesity in adolescents. Exploratory analyses of the current study suggest that the amount of effort to gain reward and to avoid punishment may play a role in the development and maintenance of overweight and obesity. However, since the effort measure was a construct based on face validity and has not been properly validated, more studies are necessary before firm conclusions can be drawn.

## Introduction

Overweight and obesity are growing problems in today’s society. Both increase the risk of developing several diseases, and are related to lower life satisfaction [1]. Between 1980 and 2008, the global prevalence of overweight increased by almost 40% and the prevalence of obesity by nearly 100% [2]. Although overweight and obesity are less common in adolescents than in adults, studies indicate that the prevalence of overweight and obesity in this group is increasing even faster than in adults [3,4]. Since more than 80% of obese adolescents will become obese adults [5], it is important to intervene at a young age. However, current interventions aimed at weight reduction suffer from high rates of drop-out [6] and relapse after weight loss [7,8]. Enhancing insight into the factors underlying the development and maintenance of overweight and obesity in adolescents represents a crucial starting point for improving existing interventions. In the current study we focus on attentional biases towards rewarding and punishing cues as potentially important factors.

The idea that an attentional bias to rewarding cues might be involved in overeating stems from the incentive sensitization theory, which was originally proposed as a theory of addiction [9]. According to this theory, repeated associations of substance use and the experience of reward result in the selective processing of reward-related information (i.e., attentional bias) [10]. Specific cues that predict reward (e.g., the sight of a substance) will, through associative conditioning, become salient and attention grabbing. This results in an attentional bias—craving cycle: an attentional bias for cues of substances elicits craving, and craving in turn triggers attentional biases (e.g., by increasing the salience of cues). This process is thought to increase the likelihood of the actual use of a substance, thereby playing an important role in the development and maintenance of substance use. In line with this theory there is ample evidence that individuals who use or misuse addictive substances show an attentional bias to these substances [11]. Individuals who are sensitive to rewarding cues in general might be more likely to develop an attentional bias to specific rewarding cues. Indeed, an attentional bias to general cues of reward was found to be positively related to substance use [12,13], and predictive of the increase of illicit drug use over a period of three years [14]. Drugs and food can both be considered to be rewarding substances [15], and the brain reward circuitries of substance users and overweight/obese individuals have been found to deviate from the reward circuitries of healthy controls in comparable ways [16]. Therefore, an attentional bias to rewarding cues might not only be involved in substance misuse but might also play a role in the development and maintenance of overweight/obesity. Following this, the major aim of this study is to investigate whether an attentional bias to general cues of reward is related to body mass index (BMI) in adolescence. Moreover by taking a longitudinal approach and following these adolescents for six years, it can be examined whether this attentional bias is predictive of changes in BMI. In the current study an attentional bias to general cues of reward will be indexed with a motivated game designed in the format of the spatial orientation task (SOT)[17], that was previously successfully used in the context of substance use and addiction [12–14].

An attentional bias to cues of reward has been suggested to be behavioral output of the individuals’ reward system [18,19]. This reward system, also known as the behavioral activation system, is a brain system which is thought to respond to rewarding cues in the environment with increased attention and approach behavior. Individuals who are sensitive to reward are therefore more inclined to have attention for, and respond with approach behavior in situations that are associated with appetitive or rewarding stimuli. Self-report measures of the sensitivity of this reward system have been studied in relation to overweight/obesity, yet findings are equivocal. Individuals with overweight/obesity were not found to report more sensitivity to reward than healthy weight individuals in a number of studies [20–22]. Correlational studies investigating the relationship between reward sensitivity and individuals’ BMI have shown a positive linear relationship [23], a negative linear relationship [24], and an inverted-u relationship, in which a positive relationship was found in the normal and overweight BMI range and a negative relationship in the moderate and extreme obese BMI ranges [24,25]. Methodological differences, such as the choice of questionnaire or subscale, and age differences across studies might partly explain the inconsistent findings. Yet more importantly, a limitation of previous studies is the use of self-report measures. Self-report questionnaires measure individuals’ beliefs about their behavior instead of their actual behavior. Discrepancies between these beliefs and actual behavior might arise due to, for example, individuals’ lack of insight into their own behavior, or their inability to linguistically express their own tendencies [26]. Therefore, we have decided to use a behavioral measure in the current study to examine the potential relation between sensitivity of the reward system and overweight/obesity. To address the possibility that part of the inconsistencies in previous findings may be attributed to the type of measures that were used, we complemented the behavioral measure with a self-report measure of reward sensitivity.

In addition to heightened attention to cues of reward, lowered attention to cues of punishment might also play a role in appetitive behavior. Attention to cues of punishment has been suggested to be behavioral output of an individuals’ punishment system [18,19]. This punishment system (i.e., flight-fight-freeze system), is a brain system which responds to punishing cues in the environment with increased attention and avoidance behavior [19]. A heightened sensitivity to punishment could potentially help individuals to restrict their food intake, since these individuals would be more sensitive to the aversive outcomes of overeating (e.g., becoming overweight or obese). It has been suggested that punishment sensitivity might therefore set individuals at risk for developing eating disorders such as anorexia nervosa [27,28]. In contrast, a lowered sensitivity to punishment might set individuals at risk for overeating. Although self-reported punishment sensitivity has not been found to be related to BMI [22,29], an attentional bias to general cues of punishment has not yet been studied in relation to overweight/obesity. Therefore, the second goal of the current study is to investigate the relation between an attentional bias towards cues of punishment and BMI. Also for punishment sensitivity, the behavioral measure will be complemented with a self-report measure of punishment sensitivity.

Attentional biases are considered to be automatic processes [11], and therefore unintentional. Not only these automatic processes, but also more intentional reward and punishment related processes may play a role in the development and maintenance of overweight and obesity. Therefore, the last goal of the current study is to explore whether individuals with overweight/obesity put more effort into rewarding than into punishing parts of the behavioral task. The amount of effort put into the task is thought to be more under the participants’ own control (i.e., one can choose how much effort one invests into the task). We expect that individuals who are more willing to put effort into the part of the task where they can receive a reward, than into the part where they can avoid getting punished would be at risk for overeating and thus for a heightened BMI.

To sum up, this study tests whether in a community cohort of adolescents, (1) there is a positive relation between the strength of an attentional bias to general cues of reward and BMI, and a negative relation between the strength of an attentional bias to general cues of punishment and BMI, (2) a relatively strong attentional bias to general cues of reward, and a relatively weak attentional bias to general cues of punishment, is predictive of change in BMI over a six year period, (3) self-reported reward sensitivity is positively, and self-reported punishment sensitivity is negatively related to BMI, (4) relatively high self-reported reward sensitivity and relatively low self-reported punishment sensitivity is predictive of change in BMI over a six year period, and lastly whether (5) greater effort during rewarding parts of a game compared to the effort during punishing parts of the game is positively related to BMI, and predictive of change in BMI over a six year period.

## Method

### Participants

Participants of the TRacking Adolescents’ Individual Lives Survey (TRAILS) were included in the current study. TRAILS is a large prospective population study of Dutch adolescents from five northern municipalities (both rural and urban areas) in the Netherlands. The TRAILS cohort consists of children born between 1 October 1989 and 30 September 1990 (two northern municipalities), and children born between 1 October 1990 and 30 September 1991 (remaining three northern municipalities). A total of 2230 children were included in TRAILS at baseline, which took place from March 2001 through July 2002 [30, 31]. All adolescents and their parents gave written informed consent.

The current study reports on data from the second (T2), third (T3) and fourth (T4) assessment waves [32]. During T2, which ran from September 2003 to December 2004, 2,149 adolescents participated (96.4% of the initial sample). During T3, which ran from September 2005 to August 2007, 1,816 adolescents participated (81.4% of the initial sample). T4 ran from October 2008 to September 2010, and 1,881 adolescents participated (84.3% of the initial sample). During T3 a series of laboratory tasks were performed on top of the general assessments. The spatial orientation task (SOT) was the first of these laboratory tasks. For the laboratory tasks a focus group of 744 participants was invited, of which 715 (96%) agreed to participate. Adolescents with a high risk of mental health problems were overrepresented in this focus group. High risk was defined based on temperament (high frustration and fearfulness, low effortful control), lifetime parental psychopathology (depression, anxiety, addiction, antisocial behavior or psychoses), and/or living in a single parent family. Of the focus cohort 66.2% had at least one of these risk factors. The remaining 33.8% were randomly selected from the low-risk TRAILS participants (See S1 Table for the distribution of participants across the different risk profiles and how these risk factors were measured). It is possible to represent the TRAILS distribution in this focus cohort by means of sampling weights [30].

For the current study two selections were made. First, individuals with self-reported reward and punishment sensitivity data (T 2) as well as age, height and weight on T2, T3, and T4 (to calculate adjusted-BMI) were selected (*N* = 1306, 51.2% females). Secondly, individuals who participated in the behavioral measure of reward and punishment sensitivity (T3), and with age, height and weight available on T2, T3 and T4 (to calculate adjusted-BMI) were selected. Of the initial 715 participants who participated in the laboratory tasks (including the SOT), two participants had incomplete SOT data, and of 106 participants adjusted BMI at T 2, T3, and/or T4, could not be calculated for, resulting in a sample of 607 participants (53.8% females). In summary, for the analyses on the self-report data we will report on 1,306 participants, and for the behavioral measure we will report on data of 607 participants. Table 1 gives an overview of the characteristics of the samples.

**Table 1:** Sample characteristics.

*Self-report sample (N = 1306)*			
	**M (SD) or percentage**		
	**T2**	**T3**	**T4**
Age	13.51 (0.52)	16.21 (0.66)	19.00 (0.57)
Adjusted BMI	101.00 (15.82)	105.32 (15.61)	108.47 (17.94)
Behavioral measure sample (N = 610)*			
	**M (SD) or percentage**		
	**T2**	**T3**	**T4**
Age	13.49 (0.51)	16.13 (0.59)	18.95 (0.53)
Adjusted BMI	101.29 (16.08)	105.90 (15.98)	109.26 (18.87)

*Note*.

* The sample size reported reflects the weighted sample size. Adjusted BMI = ((actual BMI/Percentile 50 of BMI for age and gender) x 100).

### Measures

#### Body Mass Index

At each assessment wave height and weight of the participants were measured by means of a standardized measurement procedure. At T4, four participants had self-reported weight, and one participant had self-reported height and weight. Since BMI (weight/height^2^) in children changes substantially with age, an age related cut-off score is necessary to make BMI of adolescents comparable to each other and comparable over several years [33]. Therefore, the adjusted BMI was calculated ((actual BMI/Percentile 50 of BMI for age and gender) x 100). The 50^th^ percentile of BMI for age and gender was obtained from the Netherlands Organization for Applied Scientific Research [34]. Adjusted BMI scores between 85% and 120% were considered as normal weight, smaller than 85% as underweight, larger than 120% as overweight, and larger than 140% as obese [35]. Table 2 shows the prevalence of underweight, normal weight, overweight, and obesity in the self-report and behavioral measure sample, during T2, T3, and T4.

**Table 2:** Prevalence of underweight, overweight and obese during wave 2, wave 3 and wave 4.

*Self-report (BIS/BAS) sample (N = 1306)*			
	**T2**	**T3**	**T4**
Underweight	10.2%	2.8%	2.6%
Normal weight	80.0%	84.5%	78.8%
Overweight	7.0%	9.6%	13.0%
Obese	2.8%	3.1%	5.6%
Behavioral measure (SOT) sample (N = 610)*			
	**T2**	**T3**	**T4**
Underweight	11.4%	3.0%	2.8%
Normal weight	77.5%	83.7%	78.2%
Overweight	8.4%	10.0%	12.3%
Obese	2.7%	3.3%	6.8%

*Note*.

* The sample size reported reflects the weighted sample size.

#### Self-reported reward and punishment sensitivity

The Dutch translation of the BIS/BAS was used [36]. This questionnaire measures reward (BAS) and punishment (BIS) sensitivity. The BAS has three subscales; drive, fun seeking, and reward responsiveness. The questionnaire has 20 items which are scored on a four-point Likert scale from *“very true for me”* to *“very false for me”*. In the current study the BIS (e.g., “Criticism or scolding hurts me quite a bit”), which consists of eight items, the BAS- reward responsiveness (e.g., “When I am doing well at something, I love to keep at it”), which consists of five items, and the BAS- drive (e.g., “I go out of my way to get things I want”), will be reported. Subscale scores are the average scores of the relevant items. For individuals who missed a maximum of one item per subscale the mean score of the subscale was still calculated based on the remaining items (less than 1.0% of the cases). There was one participant who missed more than one item and was therefore excluded from the analyses. The subscales had acceptable internal reliability as is often found for the BIS/BAS subscales (Cronbach's alpha of 0.68, 0.62, and 0.63 respectively).

#### Spatial Orientation Task

The SOT [17] was developed to explore to what extent individuals direct and hold their attention to places of reward or punishment. It represents a multifaceted task in which participants respond to single targets with a button press (the “b” key on the keyboard) as soon as they detect it. The target is preceded by a valid or invalid cue (displayed for 250 ms (short delay), or 500 ms (long delay)), which has predictive value for the chance of a positive (reward or non-punishment) or a negative (punishment or non-reward) outcome. All different facets of the task will be discussed in detail below.

Procedure. Participants performed the task on an Intel Pentium 4 CPU computer with a Philips Brilliance 190 P monitor. The task was run by E-prime software version 1.1 (Psychology Software Tools Inc., Pittsburgh, Pennsylvania). Participants were seated 50 cm away from the screen and responses were collected on the computer’s keyboard.

Throughout the task two black bars were displayed, one on the right and one on the left side of the screen, against a white background. These bars marked the location of the cue and the target. In between these two bars (i.e., in the middle of the screen) the current score was shown in black throughout each trial. Participants were instructed to focus on this score during the game. Signaling the start of a new trial, the current score disappeared from the screen for 200 ms after which it reappeared. After a 250 ms delay, a cue (see Stimuli) replaced one of the two black bars. Then, after a delay of either 250 or 500 ms, the target (a small grey rectangle) appeared either centered within the cue (cued trial), or centered within the remaining black bar on the other side of the screen (uncued trial). In some trials no target appeared (catch trials), and participants were supposed not to respond with a button press during these trials. The presentation of the cue for either 250 or 500 ms provides the opportunity to examine the relative importance of both automatic and more voluntary attentional processes [17]. After 500 ms (or 1000 ms on catch trials) participants were presented with a feedback signal (see Stimuli) below the score in the middle of the screen and the two black bars were reinstated removing the cue and the target from the screen. After another 250 ms the score was updated (if changed) (see Scoring) and the new trial started after a randomly selected intertrial interval of either 500 or 1000 ms. The score was set to zero at the beginning of each block (See S1 Fig and [13]).

The task consisted of two different types of blocks. During winning games participants could win 10 points on trials at which they responded sufficiently fast (see Scoring) and did not gain points on trials at which they responded too slow. During losing games participants could lose 10 points on trials on which they responded too slow and did not lose points on trials on which they responded sufficiently fast. Regardless of the block, participants would lose 10 points if they responded inaccurately (i.e., before the target was shown or on catch trials). Furthermore, participants were told at the beginning of the task that those with the highest scores in the winning games would win an attractive prize (e.g., a balloon ride) and that having an extremely low score on the losing games would result in having to redo the task until performance was good enough. All participants started with two winning games, then continued with two losing games followed by another two winning and two losing games. Each block consisted of 32 cued trials (57%), 16 uncued trials (29%) and 8 catch trials (14%) in random order. The test blocks were preceded by a winning and a losing practice block, which consisted of 6 cued, 6 uncued, and 2 catch trials.

Stimuli. The task includes two different cues that can precede the target; a blue arrow pointing upwards and a red arrow pointing downwards. Participants were told that the blue cue would signal that responding fast to the target appearing in that location would be easy since it would result in a fast enough response 75% of the time. Responding fast to the target in the uncued location in such a trial however would be hard and would result in an insufficiently fast response 75% of the time. Furthermore, participants were told that the red cue would signal that responding fast to the target appearing in that location (cued trial) would be hard since it would result in an insufficiently fast response 75% of the time. Whereas responding fast to the target when it appeared in the location opposite to the cue would be easy since it would result in a sufficiently fast response 75% of the time. Additionally, participants were informed that cues indicated the probable location of the target, with 2/3 of the targets appearing in the cued location. Thus, in general the blue cue was a signal for a high chance of a fast enough response, and the red cue was a signal for a high chance of a too slow response. Lastly, participants were informed that there occasionally would be trials where no target appeared.

Both the blue upward arrow and the red downward arrow were used as a feedback signal as well. Here, the blue arrow pointing upwards signaled a fast enough response on targeted trials or a correct nonresponse on catch trials. The red arrow pointing downward signaled a too slow response on targeted trials or an inappropriate response on catch trials.

Scoring. At the end of each game the participant’s median reaction time and standard deviation was calculated to compute cutoffs for fast and slow responses in the following game of the same type. For the first two practice blocks a fixed cutoff of 350 ms was used since no personalized cutoffs were available for these blocks. During easy trials (cued blue or uncued red) responses were labeled sufficiently fast when they were faster than participant’s median reaction time plus 0.55 times the standard deviation. During hard trials (uncued blue or cued red) responses were labeled sufficiently fast when they were faster than participant’s median reaction time minus 0.55 times the standard deviation. Further, since reaction times tend to be about 25 ms slower after a short cue delay time then after a long cue delay time [17], 12 ms were added to the median reaction time for short-delay trials and 12 ms were subtracted from the median reaction time for long-delay trials (See Table 3 for an overview).

**Table 3:** Overview of trials of the spatial orientation task.

Cue	Target	Odds	Cue delay time	Cutoff for fast response^1^	Correction for cue delay time	Anticipated outcome
Blue	Cued	2/3	250 ms	Median RT + 0.55 SD	+ 12 ms	75% chance of **positive** outcome
	Cued	2/3	500 ms	Median RT + 0.55 SD	– 12 ms	75% chance of **positive** outcome
	Uncued	1/3	250 ms	Median RT– 0.55 SD	+ 12 ms	75% chance of **negative** outcome
	Uncued	1/3	500 ms	Median RT– 0.55 SD	– 12 ms	75% chance of **negative** outcome
Red	Cued	2/3	250 ms	Median RT– 0.55 SD	+ 12 ms	75% chance of **negative** outcome
	Cued	2/3	500 ms	Median RT– 0.55 SD	– 12 ms	75% chance of **negative** outcome
	Uncued	1/3	250 ms	Median RT + 0.55 SD	+ 12 ms	75% chance of **positive** outcome
	Uncued	1/3	500 ms	Median RT + 0.55 SD	– 12 ms	75% chance of **positive** outcome

*Note*. RT = reaction time ^1^ Since the cutoff score is calculated relative to performance, this is not expected to influence performance of some individuals differently than performance of others.

### Procedure

**Fig 1:**
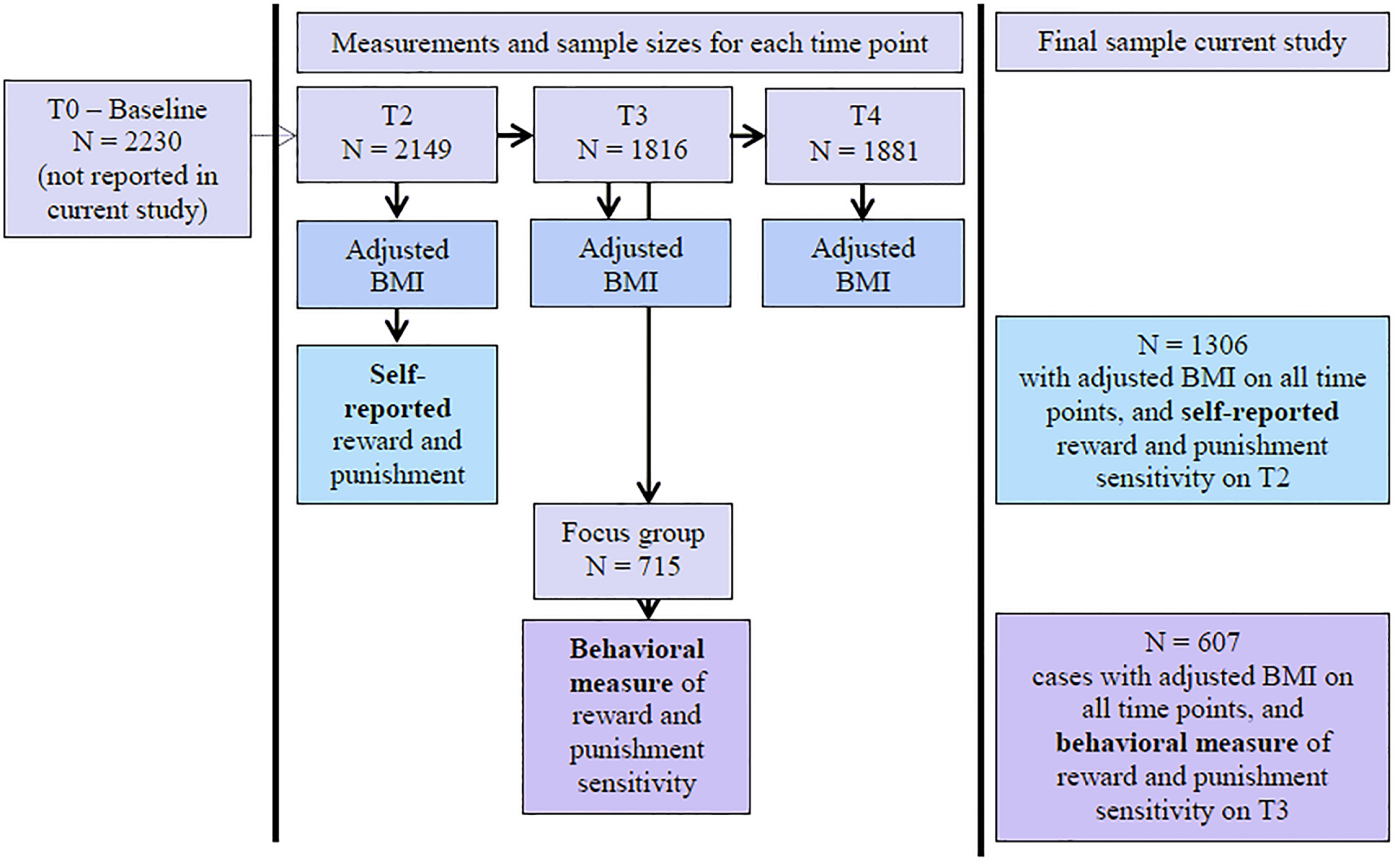
Study design and participant flow.

The current study reports on data from a large prospective cohort study, and within the current study a cross-sectional as well as a longitudinal approach were taken. The study was approved by the Central Committee on Research Involving Human Subjects (CCMO). Length and weight were measured during the regular assessments, which took place at the TRAILS offices. The BIS/BAS questionnaire was part of the regular assessment taking place at the TRAILS offices at T2. Participants of the laboratory tests were tested at selected locations in their town of residence, in a sound-attenuating room with blinded windows. In order to optimize standardization of the experimental session, test-assistants received extensive training. A detailed overview of the procedure is provided in Fig 1.

### Data reduction attentional biases

The SOT data were analyzed following van Hemel-Ruiter et al. [13,14], who analyzed these data in their study on reward related-attentional bias and substance use. We differentiated between the winning and losing games of the SOT in the current study, because sensitivity for reward might differ in its relevance for overweight/obesity from non-punishment, as might sensitivity to punishment from non-reward. Furthermore, reward was emphasized during the winning games (possibility to win points during the game and to win an attractive prize), and punishment was emphasized during the losing games (possibility to lose points during the game and to have to redo the task until performance is good enough). Thus at the start of the game the reward cue was already a more salient cue than the non-punishment cue, and the punishment cue a more salient cue than the non-reward cue. Therefore, the reaction times of the winning games were used as measurement of attentional bias to reward, and reaction times during the losing games as measurement of attentional bias to punishment.

Individuals are found to respond faster to targets that appear in a location to which they are attending to [37]. Therefore, facilitated engagement to reward is inferred when on winning games (emphasis on reward) participants responded faster to targets that appeared in a location that was preceded by a blue cue (signaling high chance of reward) than in a location that was preceded by a red cue (signaling a high chance of non-reward). Thus, when they attended more to rewarding than to non-rewarding cues. Analogously, facilitated engagement to punishment is inferred when on losing games (emphasis on punishment) participants responded faster to targets that appeared in a location that was preceded by a red cue (signaling high chance of punishment) than to targets that appeared in a location that was preceded by a blue cue (signaling a high chance of non-punishment). Thus when they attended more to punishing than to non-punishing cues.

Difficulty to disengage from reward is inferred when participants had more difficulty to look away from rewarding cues than from non-rewarding cues. Thus, when during winning games, individuals responded slower to uncued targets preceded by a blue cue (signaling high chance of reward) than to uncued targets preceded by a red cue (signaling high chance of non-reward). Analogously, difficulty to disengage from punishment is inferred when participants had more difficulty to look away from punishing cues than from non-punishing cues. Thus, when on losing games individuals were faster to respond to uncued targets preceded by a red cue (signaling high chance of punishment) than to uncued targets preceded by a blue cue (signaling high chance of non-punishment). The attentional bias scores were calculated according to these derivations, separately for the short (250 ms) and long (500 ms) cue delay time (see Table 4).

**Table 4:** Calculation of attentional biases to reward and punishment.

Game	Bias	Calculation	Interpretation	Cue delay time	
Winning	Attentional	mean RT cued red trials –	high score = high	250 ms	Automatic
game	engagement	mean RT cued blue trials	AB to reward	500 ms	Voluntary
	Difficulty to	mean RT uncued blue trials–	high score = high	250 ms	Automatic
	disengage	mean RT uncued red trials	AB to reward	500 ms	Voluntary
Losing	Attentional	mean RT cued blue trials–	high score = high	250 ms	Automatic
game	engagement	mean RT cued red trials	AB to punishment	500 ms	Voluntary
	Difficulty to	mean RT uncued red trials–	high score = high	250 ms	Automatic
	disengage	mean RT uncued blue trials	AB to punishment	500 ms	Voluntary

*Note*. RT = reaction time, AB = attentional bias.

Furthermore, we exploratory examined whether a relative measure of overall speed of responses on the winning games compared to losing games was related to BMI. Overall speed was calculated by averaging reaction times of all trials (cued, uncued, short cue delay time, and long cue delay time) of the winning and losing games separately. The mean reaction time of the winning games then was subtracted from the mean of the losing games, providing the difference in effort participants put in the winning and losing games. Higher scores on this measure reflect relatively high effort during the games with potential reward. Since the speed of the responses might change over the course of the game, for example due to a learning effect or decreased motivation, effort scores were calculated separately for the first half and the second half of the game.

### Statistical analyses

1. Firstly, the relation between the attentional bias indices and BMI was examined. As a first step bivariate correlation analyses were performed to see whether attentional bias measures correlate with adjusted BMI at T2, T3, T4, and with the adjusted-BMI-change variables. Change in adjusted BMI was calculated by subtracting adjusted BMI at T2 from adjusted BMI at T3; T3 from T4, and T2 from T4. To test whether attentional biases to reward and punishment cues were related to BMI, or to the change in adjusted BMI, a multiple regression analysis was performed with BMI as dependent variable and attentional bias scores as independent variables.
2. Secondly, the relation between self-reported reward and punishment sensitivity and BMI was examined. Again, as a first step bivariate correlational analyses were performed to see whether BIS, and the subscales BAS-Reward responsiveness and BAS-Drive are related to adjusted BMI and change in adjusted BMI. To test whether self-report reward and punishment sensitivity are related to BMI, or change in adjusted BMI, a multiple regression analysis was performed with BMI as dependent variable and BIS, BAS-Reward responsiveness and BAS-Drive as independent variables.
3. Lastly, we examined the relationship between the effort score and BMI. Bivariate correlation analyses were performed to examine whether these effort scores (of the first and second half separately) correlate with adjusted BMI and change in adjusted-BMI. To test whether the effort scores indeed represent relatively independent constructs, bivariate correlational analyses were performed between the effort scores and the attentional bias scores.

## Results

### Descriptive statistics

BMI. A bivariate correlational analysis on adjusted BMI and adjusted BMI change scores (See S2 Table) showed that individuals with a relatively high BMI at T2, had a relatively high BMI at T3 and T4 (*r* = 0.84, p < 0.001 and *r* = 0.75, *p <* 0.001), and individuals with a relatively high BMI at T3 also had a relatively high BMI at T4 (*r* = 0.86, *p* < 0.001). Individuals with a relatively high increase in BMI between T2 and T3, and T2 and T4, had a relatively low BMI at T2 (*r* = -0.30, p < 0.001 and *r* = -0.20, *p* < 0.001). Furthermore, individuals with a relatively high increase in BMI between T3 and T4, had a relatively high BMI at T4 (*r* = 0.49, *p* < 0.001), whereas BMI at T2 and T3 did not predict change in BMI between T3 and T4.

SOT. Trials on which participants responded before the target appeared were removed. This resulted in the deletion of 8.3% of the trials. Trials during which participants did not respond to the target were also deleted. This resulted in an additional deletion of 3.3% of the trials. Lastly, reaction times below 125 ms, which were most likely anticipation errors, were deleted [13]. This resulted in deletion of an additional 8.5% of the remaining trials. The mean reaction times for each trial type (easy cue/hard cue & cued/uncued) and game type (positive & negative) were calculated after deletion of these outliers, and are shown in Table 5.

**Table 5:** Mean reaction times and standard deviations of the Spatial Orientation Task.

	Cued		Uncued	
	Blue	Red	Blue	Red
**Short cue delay time (250 ms)**				
**WG**	333 (40)	364 (47)	465 (89)	467 (88)
**LG**	326 (44)	355 (51)	453 (87)	455 (93)
**Long cue delay time (500 ms)**				
**WG**	340 (57)	377 (67)	380 (76)	375 (72)
**LG**	329 (58)	363 (68)	378 (80)	371 (75)

*Note*. *N* = 610. The sample size reported reflects the weighted sample size. WG = winning game, LG = losing game.

Table 6 shows the mean reaction times on winning games, the mean reaction time on losing games, and the mean effort score for the complete game, and the first and second half of the game separately. A paired t-test showed that in general participants were faster on losing games than on winning games (*95% CI* [-11.23; -7.97]). This difference was also found when investigating the first half of the game (*95% CI* [-15.75; -10.97]), and the second half of the game (*95% CI* [-7.93; -3.75]) separately. Correlational analyses to test whether effort and attentional bias indeed represent relatively independent constructs showed that effort during the first half of the game was negatively related to attentional engagement to punishment on the long delay trials (*r* = -0.11, *p* <0.01), but not related to any of the other attentional bias measures. Effort during the second half of the game was not related to any of the attentional bias measures.

**Table 6:** Mean reaction times on positive and negative games, and effort scores of the Spatial Orientation Task, and separately for the first and the second half of the task.

		RT	SD
**Complete task**	**Winning games**	375.54	48.72
	**Losing games**	365.94	48.60
	**Effort (Losing–Winning)**	-9.60	20.45
**First half of the task**	**Winning games**	391.76	51.26
	**Losing games**	378.40	52.35
	**Effort (Losing–Winning)**	-13.36	30.02
**Second half of the task**	**Winning games**	359.32	51.63
	**Losing games**	353.49	49.93
	**Effort (Losing–Winning)**	-5.84	26.28

*Note*. N = 609, The sample size reported reflects the weighted sample size.

### Task design check

Participants were faster on cued blue than cued red trials for both winning and losing games irrespective of the cue delay time (Table 7). This reflects a general engagement effect; a preference to direct attention to cues that predict reward or non-punishment compared to cues that predict punishment and non-reward. Thus, in line with the task design, participants showed a generally enhanced attentional engagement to stimuli signaling reward and non-punishment. Furthermore, participants were slower on uncued blue trials than uncued red trials on long cue delay time trials, indicating a general difficulty to disengage from reward and non-punishment. This difference was not found for short cue delay time trials. Thus, participants were generally faster to disengage from cues predictive of punishment and non-reward than from cues predictive of reward and non-punishment when they had more time to control their attention (long cue delay time trials; Table 7).

**Table 7:** Differences between blue and red cue trials, separately for all trial types (losing vs. winning game, cued vs. uncued, short delay vs. long delay).

			99% Confidence Interval of the Difference		
		Calculation	Lower bound	Upper bound	p
**Short cue delay time (250 ms)**					
**WG**	Attentional engagement	Cued red–cued blue	27.60	34.43	< 0.001*
	Difficulty to disengage	Uncued red–uncued blue	-4.35	8.57	0.398
**LG**	Attentional engagement	Cued red–cued blue	25.06	32.30	< 0.001*
	Difficulty to disengage	Uncued red–uncued blue	-3.94	9.74	0.274
**Long cue delay time (500 ms)**					
**WG**	Attentional engagement	Cued red–cued blue	31.40	41.49	< 0.001*
	Difficulty to disengage	Uncued red–uncued blue	-11.21	0.35	0.016
**LG**	Attentional engagement	Cued red–cued blue	28.59	39.32	< 0.001*
	Difficulty to disengage	Uncued red–uncued blue	-13.08	-0.58	0.005*

*Note*. *N* = 610. The sample size reported reflects the weighted sample size, WG = winning game, LG = losing game

* α < .01 corrected for multiple tests.

### (1) Relation between attentional biases to cues signaling reward and punishment and adjusted BMI and change in adjusted BMI over time

None of the attentional bias scores were related to adjusted BMI at T2, T3 or T4, nor to changes in adjusted BMI over time (all *r <* 0.06; *p* > 0.16). Since an increase in BMI for underweight participants could be considered healthy behaviour, and the processes underlying this increase in BMI might differ from the processes that are related to becoming overweight/ obese, the analyses was repeated after excluding participants who were underweight (*N* = 80). Yet, excluding these cases did not change results. A regression analysis to test whether the attentional bias scores predict BMI or BMI change was therefore not executed.

Lastly, bivariate correlation analyses were performed separate for boys and girls to ensure that the lack of relation was not due to gender differences. For girls no relation was found between the attentional bias scores and Adjusted BMI at T2, T3 or T4, nor the changes in adjusted BMI over time. For boys engagement towards punishment on the short delay trials was significantly related to change in BMI between T2 and T3 (*r =* -0.17; *p* < 0.01). Yet, after performing a correction for multiple testing this finding did not reach significance. Additionally, it does not seem to be a robust finding since engagement towards punishment on the short delay trial was not related to change in BMI between T3 and T4 or between T2 and T4.

### (2) Relation between self-report measures of reward and punishment sensitivity and adjusted BMI and change in adjusted BMI over time

None of the adjusted BMI, or adjusted BMI change variables were related to the reward or punishment sensitivity measures (all *r <* 0.05; *p* > 0.10). Excluding underweight participants did not change this finding, nor were there gender differences. A regression analyses to test whether self-reported reward or punishment sensitivity predicts BMI or BMI change was therefore not executed.

### (3) Relation between effort on rewarding versus punishing games in relation to adjusted BMI and change in adjusted BMI over time

Table 8 shows that a higher BMI at T2, T3, and T4 was positively related to effort during the first half of the game. Thus, during the first half of the game, individuals with a higher BMI showed a relatively large difference in speed between the *winning* games and losing games. Additionally, effort during the first half of the game was significantly related to change in adjusted BMI between T2 and T3 (*r* = 0.08, *p* < 0.05), and marginally significantly related to change in adjusted BMI between T2 and T4 (*r* = 0.08, *p* = 0.06). No significant relation was found between effort during the first half of the game and change in adjusted BMI between T3 and T4. Effort during the second half of the game was negatively related to change in adjusted BMI between T3 and T4 (*r* = -0.08, *p* < 0.05), and marginally significantly related to change in adjusted BMI between T2 and T4 (*r* = -0.08, *p* = 0.05).

**Table 8:** Bivariate correlations of adjusted-BMI and self-report reward and punishment sensitivity.

	AdjustedBMI T2	Adjusted BMI T3	Adjusted BMI T4	BMI change T3-T2	BMI change T4-T3	BMI change T4-T2
Effort first half	0.09*	0.13**	0.12**	0.09*	0.02	0.08
Effort second half	0.02	-0.01	-0.05	-0.02	-0.08*	-0.08

*Note*. N = 609, The sample size reported reflects the weighted sample size.

* p < 0.05

** p < 0.01.

The relation between effort and BMI could be due to relatively fast responses during the winning games and/or relatively slow responses during the losing games. Therefore, we subsequently computed bivariate correlations between BMI and the mean reaction times on winning and losing games separately. BMI at T2, T3, and T4 were significantly related to response speed during losing games of the first half of the game (*r* = 0.08, *p* < 0.05; *r* = 0.10, *p* < 0.02 & *r* = 0.13, *p* < 0.01, respectively), but not to response speed during winning games (*r* = 0.03, *p* = 0.44; *r* = 0.03, *p* = 0.52 & *r* = 0.06, *p* = 0.15). Thus, the positive association between effort score during the first part of the game and adjusted BMI at T2, T3 and T4 seems to be explained by relatively slow responses during losing games. Increase in adjusted BMI between T2 and T4 was also associated with slow responses during losing games (*r* = 0.09, *p* < 0.02), but not with fast responses during winning games (*r* = 0.05, *p* = 0.22). Change in BMI between T2 and T3 was not significantly related to reaction times during winning or losing games (*r* = 0.04, *p* = 0.31 & *r* = -0.01, *p* = 0.79, respectively). Change in adjusted BMI between T3 and T4 was significantly related to relatively fast responses during the winning games (*r* = 0.11, *p* < 0.01), and not to the speed of responses during the losing games (*r* = 0.07, *p* = 0.09) during the second part of the game.

## Discussion

This study investigated the relationship between attentional biases towards general cues of reward or punishment and overweight/obesity. In addition, we investigated the relationship between self-reported reward and punishment sensitivity and overweight/obesity. Finally, we explored the relationship between overweight/obesity and a performance measure indicating the amount of effort invested into obtaining reward and avoiding punishment. The major results of this study can be summarized as follows: in a community cohort of adolescents, (1) attentional biases for general cues of reward and punishment (attentional engagement and difficulty to disengage) were not related to BMI, or to change in BMI over time; (2) self-reported reward and punishment sensitivity were not related to BMI, or change in BMI over time; and (3) a negative relation was found between BMI and the effort that was put in the games with a high chance of receiving punishment.

The first aim of the current study was to investigate the relation between an attentional bias to general cues of reward and BMI. The findings did not corroborate our hypothesis that a relatively strong attentional bias to general cues of reward would be related to a relatively high BMI, or to a relatively large increase in BMI over the six years follow up. An additional aim was to enhance our insight into the relation between self-reported reward sensitivity and BMI since earlier findings were inconsistent, ranging from no relation to a positive, negative, or an inverted-u relationship [23–25]. In the current study self-reported reward sensitivity (reward responsiveness as well as reward drive) was also not related to the onset or maintenance of overweight and obesity. Thus, the current findings cast doubt on the relevance of attentional bias to general cues of reward and self-reported reward sensitivity as factors in determining BMI in adolescence.

A potential methodological explanation for not finding a relation between our behavioral measure and BMI might be that the SOT [17] is not an appropriate measure of reward related attention. However, the SOT is a validated task, and attentional biases as indexed with the SOT have been linked to reward related processes before [17]. Furthermore, task performance was in line with the design of the task, and the attentional biases, as indexed by the SOT, have been shown to be sensitive to individual differences in for example alcohol and drug use [12–14]. Additionally, not only with the behavioral measure, but also with the self-report measures no relationship was found. Thus, the chosen operationalization does not seem to be the most plausible explanation for the absence of a relationship between general reward sensitivity and BMI. Another potential explanation for not finding a relationship could be that there was too little power to find an effect. However, the power of the analyses on the behavioral measure (i.e., with the smallest sample) was already very high (71% for finding very small effects), and even when manually increasing the power to 93% (to find small effect, i.e., *r =* 0.10) no significant results were found. In other words, we can conclude with a high level of certainty that an attentional bias to general cues of reward, and self-reported reward sensitivity, are not related to BMI or the change in BMI over six years in an adolescent sample.

The reward circuitries of substance users and overweight/obese individuals have been found to deviate from the reward circuitry of healthy controls in comparable ways (e.g., 16). An attentional bias to general cues of reward, and self-reported reward sensitivity were found to be positively related to substance use [13,14,38]. Yet, in the current study we did not find such a relationship between attentional bias to reward, or self-reported reward sensitivity, and BMI. One explanation could be that increased attention for general cues of reward and/or self-reported reward sensitivity are indeed related to overeating, but that compensatory behaviors blurred the relationship between attentional biases, and self-reported reward sensitivity, and BMI. Studies on the relation between addiction and reward sensitivity use substance use as outcome measure. However, studies on the relation between overeating and reward sensitivity typically rely on BMI as the long-term effect of overeating. Although BMI is of course largely influenced by individuals' eating behavior, overeating is not the sole predictor of one's future BMI. Overweight/obesity typically results from an imbalance between energy intake and energy usage [39]. This implies that changes in energy usage (e.g. by exercise) also influences BMI. In addition, genetic make-up and metabolic deviations impact BMI [40]. Therefore, it might be that an attentional bias to general cues of reward, or self-reported reward sensitivity, is related to overeating, while this is not reflected in BMI due to other factors (independent of reward sensitivity) that also have an impact on BMI. This would imply that even though the attentional bias to general cues of reward might be related to overeating, it is not crucial for the development of overweight and obesity. In line with this explanation, a prior study showed that high and low reward sensitive children indeed differed in eating behavior, but not in BMI [41].

The present study also showed that attentional bias to general cues of punishment and self-reported punishment sensitivity were not related to BMI, or change in BMI over six years. This finding was not in line with our expectation that overweight individuals would direct less attention to cues of punishment, and report less sensitivity to punishment. Rather, the current findings seem to indicate that an attentional bias to general cues of punishment and general punishment sensitivity are not related to the development or maintenance of overweight/obesity. Sensitivity to punishing cues has not received much attention in research on the development and maintenance of overweight/obesity. The two studies conducted thus far relied on self-reports and failed as well to find a relationship between punishment sensitivity and BMI [22,29]. Yet, when healthy weight individuals (mean BMI = 22.32) were compared with obese individuals (mean BMI = 30.84) and individuals with binge eating disorder (mean BMI = 38.71) separately, it was found that obese individuals reported lower levels of punishment sensitivity, and individuals with binge eating disorder higher levels of punishment sensitivity than the healthy weight participants [22]. Since both the obese individuals and the binge eating disorder patients had a higher BMI than the healthy weight participants, differences in punishment sensitivity were potentially masked in the correlational analyses. This implies that even though the attentional bias to general cues of punishment might be related to specific forms of overeating, it does not play a crucial role in the development and maintenance of overweight and obesity in general.

Finally, we explored whether the relative effort invested into the winning compared to the losing games was related to BMI. This effort score is assumed to be an index of a more deliberate expression of reward and punishment sensitivity. As expected, the exploratory analyses demonstrated that individuals with a higher BMI showed on average greater differences in reaction time speed between the winning and the losing games than individuals with a lower BMI. This difference seemed to be mainly due to individuals with a higher BMI responding slower on losing games than individuals with a lower BMI. Thus, it seems that individuals with a higher BMI were less inclined to work hard to avoid potential punishment. This might mean that individuals with a higher BMI are less influenced by potential punishment than individuals with a lower BMI. Additionally, an increase in BMI between the age of approximately 13 and 19 was related to relatively high effort during the games with a potential reward. Thus individuals who will gain in BMI over the period of six years are more willing to put effort into receive reward.

The current study has many strengths. It is the first study to investigate attentional biases to general cues of reward and punishment in the context of overweight and obesity. Further, a cross-sectional as well as longitudinal approach was taken, providing the opportunity to test the prognostic value of these attentional biases and the alleged prognostic value of self-reported reward and punishment sensitivity in the development of overweight and obesity. In addition, the sample size of the current study was relatively large which reduced the sensitivity for chance findings and enhanced the sensitivity to reliably detect even small effects. Even though the study has many strengths, there are also some limitations that should be taken into account when interpreting the results. First of all, it is important to acknowledge that the behavioral task and the self-report measure were not administered at the same time point. Thus, even though reward and punishment sensitivity are considered traits that are more or less constant, strong conclusions about the relation between the outcomes related to the behavioral measure and the self-report measure cannot be made based solely on the current study. Secondly, there was a fixed order of winning and losing games. Therefore, the cause of the larger difference in reaction time speed between the winning and the losing games should be made with caution. Additionally, since the effort measure was a construct based on face validity and has not been properly validated, more studies are necessary before firm conclusions can be drawn. It might for example be that individuals with overweight are not less motivated to avoid punishment, but freeze when there is the chance of receiving punishment.

## Conclusion

The aim of this study was to enhance insight into the characteristics underlying the development and maintenance of overweight and obesity. It was found that in adolescents, attentional biases to cues that signal reward or punishment and self-reported reward and punishment sensitivity were not related to BMI or change in BMI over six years. Although it might be that these characteristics play a role in overeating in general and binge eating specifically, these characteristics do not seem to play a crucial role in the development and maintenance of overweight and obesity in adolescents. The current study shows that individuals with a higher BMI seem to put less effort into the avoidance of punishment compared to individuals with a lower BMI, and that relatively high effort to gain reward is related to an increase in BMI between the age of 13 and 19. Future studies should further examine more intentional reward and punishment-related processes as potential factors that play a role in the development and maintenance of overweight and obesity.

## Supporting Information

S1 Fig Example of screen-setup of the Spatial Orienting Task (SOT). Example of blue cue, followed by target in the uncued location (i.e., hard target) with subsequent slow response (i.e., negative feedback). From *"* Reward-related attentional biases and adolescent substance use: The TRAILS study", by M.E. Van Hemel-Ruiter, P.J. De Jong, A. J. Oldehinkel, and B. Ostafin, 2013, Psychology of Addictive Behaviors, 27, Supplemental Material. Reprinted with permission.
(DOCX)

S1 Table Number of participants in the low and high risk profile groups in the total TRAILS population (i.e., population) and in the focus cohort of participants who performed laboratory tasks. From *"* Reward-related attentional biases and adolescent substance use: The TRAILS study", by M.E. Van Hemel-Ruiter, P.J. De Jong, A. J. Oldehinkel, and B. Ostafin, 2013, Psychology of Addictive Behaviors, 27, Supplemental Material. Reprinted with permission. *Note*. The selection criteria for high-risk profile group were as follows: High-risk temperament: EATQ (Early Adolescent Temperament Questionnaire) Frustration ≥ 90^th^ percentile or EATQ Fear ≥ 90^th^ percentile or EATQ Effortful Control ≤ 10^th^ percentile. N_A_ = 617 (27.8%), 282 girls, 335 boys. Parental psychopathology: at least one parent with severe psychopathology. N_B_ = 740 (33.3%), 393 girls, 347 boys High environmental risk: at least one of both biological parents is not part of the family. N_C_ = 526 (23.7%), 273 girls, 253 boys.
(DOCX)

S2 Table Bivariate correlations of adjusted-BMI and adjusted-BMI change variables. (DOCX)

## References

[1] Roberts RE, Hao DT. Obesity has few effects on future psychosocial functioning of adolescents. Eat Behav. Elsevier Ltd; 2013;14: 128–36. 10.1016/j.eatbeh.2013.01.008 23557808 PMC3618662

[2] WHO. Global health observatory (GHO) [Internet]. 2010 [cited 17 Oct 2013]. Available: http://www.who.int/gho/ncd/risk_factors/obesity_text/en/

[3] Onis M De, Blössner M, Borghi E. Global prevalence and trends of overweight and obesity among preschool children. Am J of Clin Nutr 2010; 1257–1264. 10.3945/ajcn.2010.29786.1 20861173

[4] Ng M, Fleming T, Robinson M, Thomson B, Graetz N, Margono C, et al Global, regional and national prevalence of overweight and obesity in children and adults 1980–2013: A systematic analysis. Lancet 2014; 384: 766–781. 10.1016/S0140-6736(14)60460-8 24880830 PMC4624264

[5] Reilly JJ, Methven E, McDowell ZC, Hacking B, Alexander D, Stewart L, et al Health consequences of obesity. Arch Dis Child. 2003;88: 748–752. 10.1136/adc.88.9.748 12937090 PMC1719633

[6] Goossens L, Braet C, Van Vlierberghe L, Mels S. Weight parameters and pathological eating as predictors of obesity treatment outcome in children and adolescents. Eat Behav. Elsevier Ltd; 2009;10: 71–3. 10.1016/j.eatbeh.2008.10.008 19171325

[7] Wilson PH. Relapse Prevention: Overview of Research Findings in the Treatment of Problem Drinking, Smoking, Obesity and Depression. Clin Psychol Psychother. 1996;3: 231–248. 10.1002/(SICI)1099-0879(199612)3:4<231::AID-CPP107>3.0.CO;2-D

[8] Poston WSC, Ericsson M., Linder J, Nilsson T, Goodrick GK, & Foreyt JP. Personality and the prediction of weight loss and relapse in the treatment of obesity. Int J Eat Disord. 1999;25: 301–309. 10191995 10.1002/(sici)1098-108x(199904)25:3<301::aid-eat8>3.0.co;2-p

[9] Berridge KC. “Liking” and “wanting” food rewards: brain substrates and roles in eating disorders. Physiol Behav. Elsevier Inc.; 2009;97: 537–50. 10.1016/j.physbeh.2009.02.044 19336238 PMC2717031

[10] Robinson TE, Berridge KC. Mechanisms of Action of Addictive Stimuli Incentive-sensitization and addiction. Addict Abingdon Engl. 2001;96: 103–14. 10.1080/09652140020016996 11177523

[11] Franken IHA. Drug craving and addiction: integrating psychological and neuropsychopharmacological approaches. Prog Neuropsychopharmacol Biol Psychiatry. 2003;27: 563–79. 10.1016/S0278-5846(03)00081-2 12787841

[12] Colder CR, O’Connor. Attention bias and disinhibited behavior as predictors of alcohol use and enhancement reasons for drinking. Psychol Addict Behav. 2002;16: 325–332. 10.1037//0893-164X.16.4.325 12503905

[13] van Hemel-Ruiter ME, de Jong PJ, Oldehinkel AJ, Ostafin BD. Reward-related attentional biases and adolescent substance use: the TRAILS study. Psychol Addict Behav. 2013;27: 142–50. 10.1037/a0028271 22564203

[14] van Hemel-Ruiter ME, de Jong PJ, Ostafin BD, Oldehinkel AJ. Reward-related attentional bias and adolescent substance use: a prognostic relationship? PLoS One. 2015;10: e0121058 10.1371/journal.pone.0121058 25816295 PMC4376386

[15] Davis C, Strachan S, Berkson M. Sensitivity to reward: implications for overeating and overweight. Appetite. 2004;42: 131–8. 10.1016/j.appet.2003.07.004 15010176

[16] Stice E, Yokum S, Burger KS. Elevated reward region responsivity predicts future substance use onset but not overweight/obesity onset. Biol Psychiatry. Elsevier; 2013;73: 869–76. 10.1016/j.biopsych.2012.11.019 23312561 PMC3774523

[17] Derryberry D, Reed MA. Anxiety-related attentional biases and their regulation by attentional control. J Abnorm Psychol. 2002;111: 225–236. 10.1037//0021-843X.111.2.225 12003445

[18] Gray J. The psychophysiological basis of introversion-extraversion. Behav Res Ther. 1970;8: 249–266. 10.1016/0005-7967(70)90069-0 5470377

[19] Gray JA, Mcnaughton N. The neuropsychology of anxiety: an enquiry into the functions of the sept-hippocampal system Oxford University Press; 2000.

[20] Schienle A, Schäfer A, Hermann A, Vaitl D. Binge-eating disorder: reward sensitivity and brain activation to images of food. Biol Psychiatry. Society of Biological Psychiatry; 2009;65: 654–61. 10.1016/j.biopsych.2008.09.028 18996508

[21] Nederkoorn C, Braet C, Van Eijs Y, Tanghe A, Jansen A. Why obese children cannot resist food: the role of impulsivity. Eat Behav. 2006;7: 315–22. 10.1016/j.eatbeh.2005.11.005 17056407

[22] Danner UN, Ouwehand C, van Haastert NL, Hornsveld H, de Ridder DTD. Decision-making impairments in women with binge eating disorder in comparison with obese and normal weight women. Eur Eat Disord Rev. 2012;20: e56–62. 10.1002/erv.1098 21308871

[23] Davis C, Patte K, Levitan R, Reid C, Tweed S, Curtis C. From motivation to behaviour: a model of reward sensitivity, overeating, and food preferences in the risk profile for obesity. Appetite. 2007;48: 12–9. 10.1016/j.appet.2006.05.016 16875757

[24] Verbeken S, Braet C, Lammertyn J, Goossens L, Moens E. How is reward sensitivity related to bodyweight in children? Appetite. Elsevier Ltd; 2012;58: 478–83. 10.1016/j.appet.2011.11.018 22138702

[25] Davis C, Fox J. Sensitivity to reward and body mass index (BMI): evidence for a non-linear relationship. Appetite. 2008;50: 43–9. 10.1016/j.appet.2007.05.007 17614159

[26] Corr PJ. Testing problems in J. A. Gray ‘ s personality theory: a commentary on Matthews and Gilliland (1999). 2001;30: 333–352.

[27] Harrison A, O’Brien N, Lopez C, Treasure J. Sensitivity to reward and punishment in eating disorders. Psychiatry Res. Elsevier Ireland Ltd; 2010;177: 1–11. 10.1016/j.psychres.2009.06.010 20381877

[28] Jonker NC, Ostafin BD, Glashouwer KA, van Hemel-Ruiter ME, de Jong PJ. Reward and punishment sensitivity and alcohol use: the moderating role of executive control. Addict Behav. 2014;39: 945–8. 10.1016/j.addbeh.2013.12.011 24389069

[29] Matton A, Goossens L, Braet C, Vervaet M. Punishment and reward sensitivity: are naturally occurring clusters in these traits related to eating and weight problems in adolescents? Eur Eat Disord Rev. 2013;21: 184–94. 10.1002/erv.2226 23426856

[30] Huisman M, Oldehinkel AJ, de Winter A, Minderaa RB, de Bildt A, Huizink AC, et al Cohort profile: the Dutch “TRacking Adolescents” Individual Lives’ Survey'; TRAILS. Int J Epidemiol. 2008;37: 1227–35. 10.1093/ije/dym273 18263649

[31] Ormel J, Oldehinkel AJ, Sijtsema J, van Oort F, Raven D, Veenstra R, et al The TRacking Adolescents’ Individual Lives Survey (TRAILS): design, current status, and selected findings. J Am Acad Child Adolesc Psychiatry. Elsevier Inc.; 2012;51: 1020–36. 10.1016/j.jaac.2012.08.004 23021478

[32] Oldehinkel AJ, Rosmalen JG, Buitelaar JK, Hoek HW, Ormel J, Raven D, et al Cohort Profile Update: The TRacking Adolescents’ Individual Lives Survey (TRAILS). Int J Epidemiol. 2015;44: 76–76n. 10.1093/ije/dyu225 25431468 PMC4339762

[33] Cole TJ, Bellizzi MC, Flegal KM, Dietz WH. and obesity worldwide: international survey. 2000; 1–6. 10.1136/bmj.320.7244.1240 PMC27365 10797032

[34] TNO. BMI-for-age charts. In: TNO Growth Charts [Internet]. 2010 [cited 26 Jan 2015]. Available: https://www.tno.nl/nl/aandachtsgebieden/gezond-leven/prevention-work-health/gezond-en-veilig-opgroeien/groeidiagrammen-in-pdf-formaat/.

[35] Van Winckel M. & Van Mil E. Wanneer is dik té dik? [When is fat too fat?] In: Braet C & Van Winckel M, editor. Behandelstrategieën bij kinderen met overgewicht [Treatment strategies in overweight children]. Houten/Diegem: Bohn Stafleu Van Loghum; 2001 pp. 11–26.

[36] Carver CS, White TL. Behavioral inhibition, behavioral activation, and affective responses to impending reward and punishment: The BIS/BAS Scales. J Pers Soc Psychol. 1994;67: 319–333. 10.1037//0022-3514.67.2.319

[37] Posner MI, Inhoff AW, Friedrich FJ. Isolating attentional systems: a cognitive-anatomical analysis. Psychobiology. 1987;15: 107–121.

[38] Bijttebier P, Beck I, Claes L, Vandereycken W. Gray’s Reinforcement Sensitivity Theory as a framework for research on personality-psychopathology associations. Clin Psychol Rev. 2009;29: 421–30. 10.1016/j.cpr.2009.04.002 19403216

[39] Wang YC, Gortmaker SL, Sobol AM, Kuntz KM. Estimating the Energy Gap Among US Children: A Counterfactual Approach. Pediatrics. 2006;118: e1721–e1733. 10.1542/peds.2006-0682 17142497

[40] Bouchard C, Trmblay A, Després JP, Nadeau A, Lupien PJ, Thériault G, et al The response to long-term overfeeding in identical twins. The New England Jounal of Medicine. 1990;332: 1477–1482. 10.1056/NEJM199005243222101 2336074

[41] Guerrieri R, Nederkoorn C, Jansen A. The interaction between impulsivity and a varied food environment: its influence on food intake and overweight. Int J Obes (Lond). 2008;32: 708–14. 10.1038/sj.ijo.0803770 18059403

