# Supplementary material for: Attentional Bias for Reward and Punishment in Overweight and Obesity: The TRAILS Study
Source: PLoS One. 2016 Jul 8;11(7):e0157573. doi: 10.1371/journal.pone.0157573 (PMC4938215; doi:10.1371/journal.pone.0157573)
Supplement: S1 Fig — Example of blue cue, followed by target in the uncued location (i.e., hard target) with subsequent slow response (i.e., negative feedback). From " Reward-related attentional biases and adolescent substance use: The TRAILS study", by M.E. Van Hemel-Ruiter, P.J. De Jong, A. J. Oldehinkel, and B. Ostafin, 2013, Psychology of Addictive Behaviors, 27, Supplemental Material. Reprinted with permission. (DOCX) [file pone.0157573.s001.docx]

B


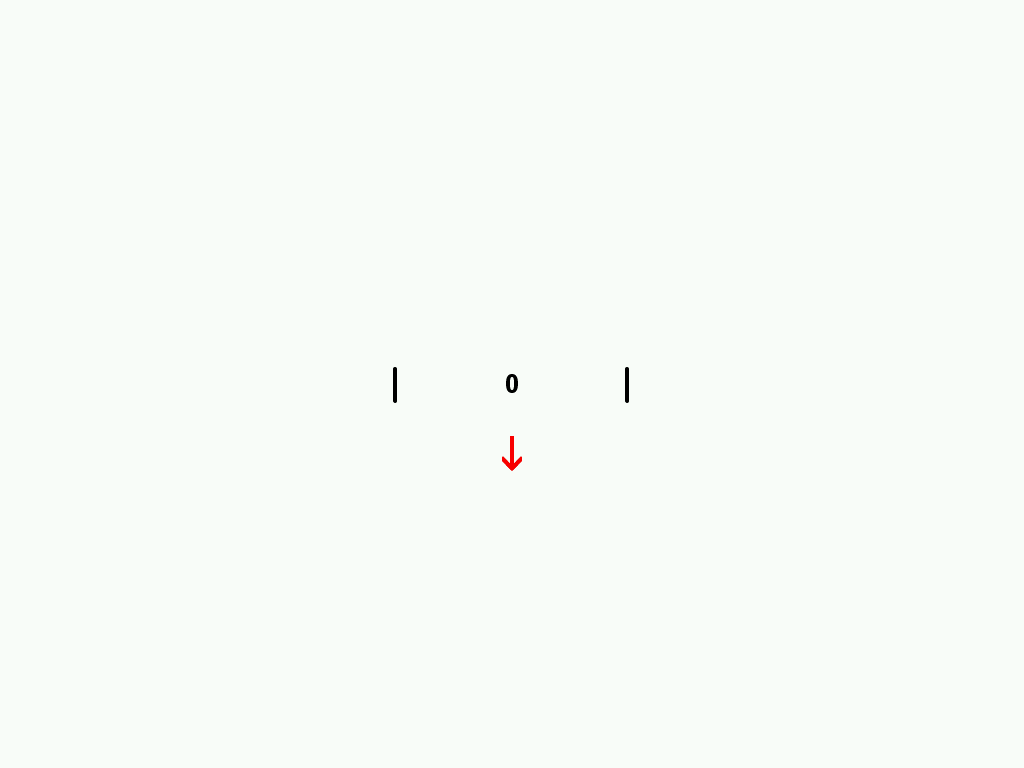


B


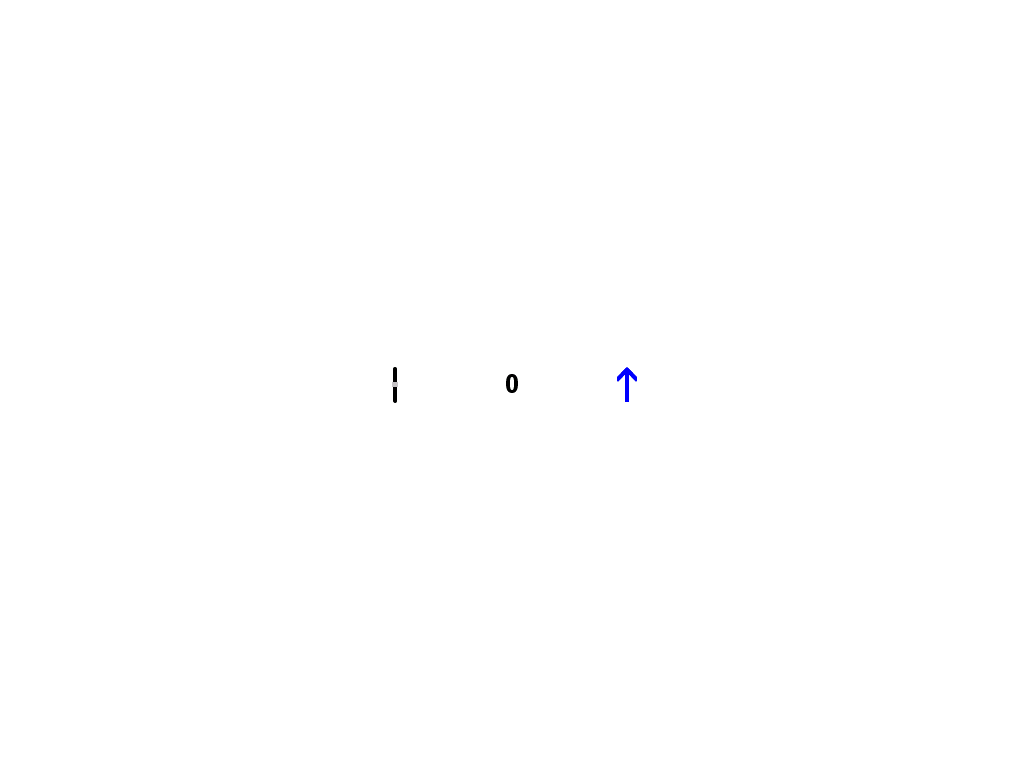


B


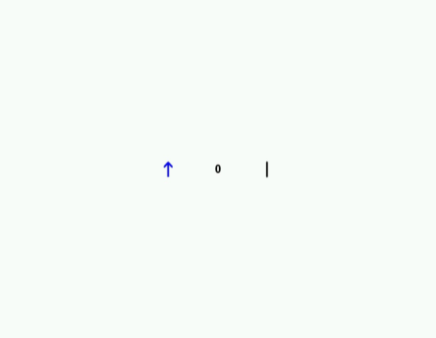


B


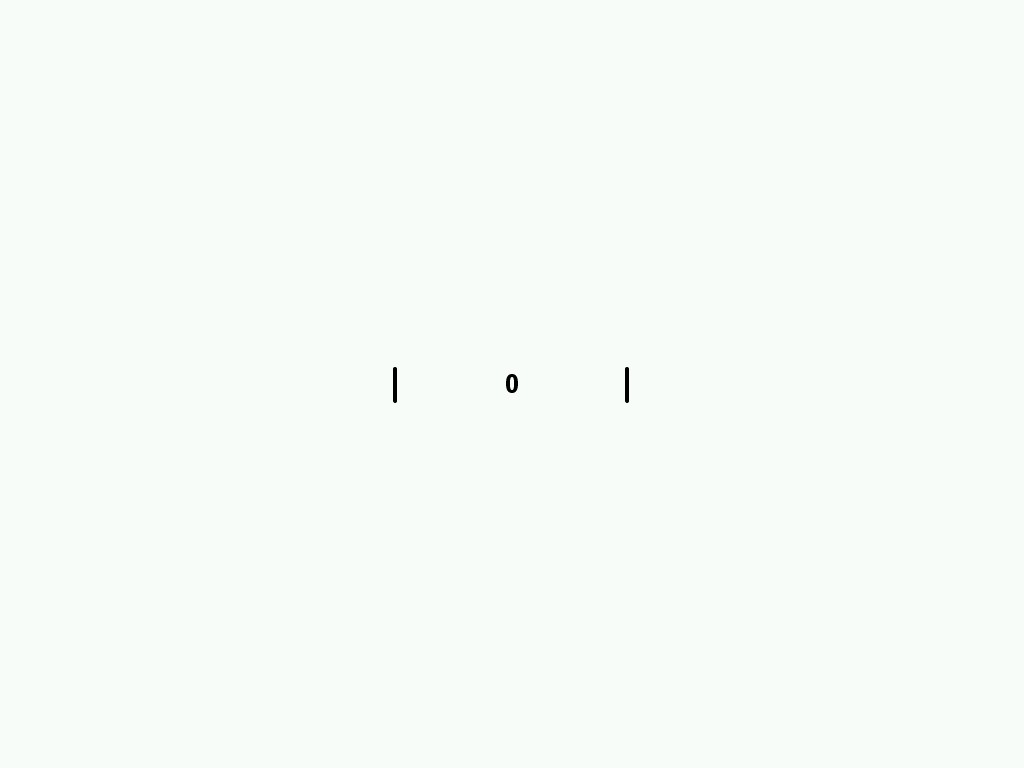


B


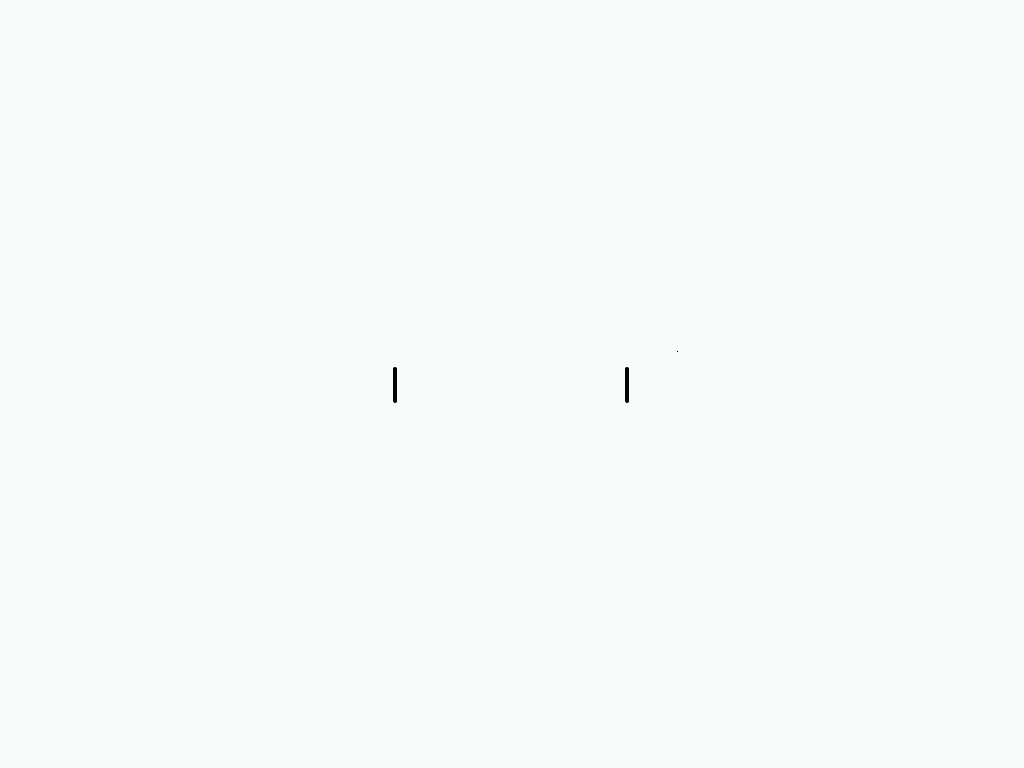


Press “B”

*S1 Figure* Example of screen-setup of the Spatial Orienting Task (SOT) - Example of blue cue, followed by target in the uncued location (i.e., hard target) with subsequent slow response (i.e., negative feedback). From *"* Reward-related attentional biases and adolescent substance use: The TRAILS study", by M.E*.* Van Hemel-Ruiter, P.J. De Jong, A. J. Oldehinkel, and B. Ostafin, 2013, Psychology of Addictive Behaviors, 27, Supplemental Material. Reprinted with permission.
