# Supplementary material for: Attentional Bias for Reward and Punishment in Overweight and Obesity: The TRAILS Study
Source: PLoS One. 2016 Jul 8;11(7):e0157573. doi: 10.1371/journal.pone.0157573 (PMC4938215; doi:10.1371/journal.pone.0157573)
Supplement: S1 Table — From " Reward-related attentional biases and adolescent substance use: The TRAILS study", by M.E. Van Hemel-Ruiter, P.J. De Jong, A. J. Oldehinkel, and B. Ostafin, 2013, Psychology of Addictive Behaviors, 27, Supplemental Material. Reprinted with permission. Note. The selection criteria for high-risk profile group were as follows: High-risk temperament: EATQ (Early Adolescent Temperament Questionnaire) Frustration ≥ 90th percentile or EATQ Fear ≥ 90th percentile or EATQ Effortful Control ≤ 10th percentile. NA = 617 (27.8%), 282 girls, 335 boys. Parental psychopathology: at least one parent with severe psychopathology. NB = 740 (33.3%), 393 girls, 347 boys High environmental risk: at least one of both biological parents is not part of the family. NC = 526 (23.7%), 273 girls, 253 boys. (DOCX) [file pone.0157573.s002.docx]

| S1 Table  *Number of participants in the low and high risk profile groups in the total TRAILS population (i.e., population) and in the focus cohort of participants who performed laboratory tasks* | | | | |
| --- | --- | --- | --- | --- |
|  |  | **Boys** | **Girls** | **Total** |
|  |  | **N** | **N** | **N** |
| Low risk (not A, B or C) | population  *focus cohort* | 462  *119* | 477  *123* | 939  *242* |
| Temperament (A) | population  *focus cohort* | 165  *53* | 138  *56* | 303  *109* |
| Parental psychopathology (B) | population  *focus cohort* | 142  *51* | 175  *52* | 317  *103* |
| Single-Parent family (C) | population  *focus cohort* | 79  *28* | 96  *38* | 175  *66* |
| A+B | population  *focus cohort* | 72  *33* | 66  *32* | 138  *65* |
| A+C | population  *focus cohort* | 41  *13* | 25  *10* | 66  *23* |
| B+C | population  *focus cohort* | 76  *31* | 99  *33* | 175  *64* |
| A+B+C | population  *focus cohort* | 57  *23* | 53  *20* | 110  *43* |
| Total | population  *focus cohort* | 1094  *351* | 1129  *364* | 2223  *715* |
| From *"* Reward-related attentional biases and adolescent substance use: The TRAILS study", by M.E*.* Van Hemel-Ruiter, P.J. De Jong, A. J. Oldehinkel, and B. Ostafin, 2013, Psychology of Addictive Behaviors, 27, Supplemental Material. Reprinted with permission.  *Note.* The selection criteria for high-risk profile group were as follows:   1. High-risk temperament: EATQ (Early Adolescent Temperament Questionnaire) Frustration ≥ 90^th^ percentile or EATQ Fear ≥ 90^th^ percentile or EATQ Effortful Control ≤ 10^th^ percentile. N_A_ = 617 (27.8%), 282 girls, 335 boys. 2. Parental psychopathology: at least one parent with severe psychopathology. N_B_ = 740 (33.3%), 393 girls, 347 boys 3. High environmental risk: at least one of both biological parents is not part of the family. N_C_ = 526 (23.7%), 273 girls, 253 boys. | | | | |
