# Supplementary material for: Attentional Bias for Reward and Punishment in Overweight and Obesity: The TRAILS Study
Source: PLoS One. 2016 Jul 8;11(7):e0157573. doi: 10.1371/journal.pone.0157573 (PMC4938215; doi:10.1371/journal.pone.0157573)
Supplement: S2 Table — (DOCX) [file pone.0157573.s003.docx]

| S2 Table  *Bivariate correlations of adjusted-BMI and adjusted-BMI change variables.* | | | | | |
| --- | --- | --- | --- | --- | --- |
|  | **1.** | **2.** | **3.** | **4.** | **5.** |
| 1. Adjusted BMI T2 | - | - | - | - | - |
| 2. Adjusted BMI T3 | 0.84** | - | - | - | - |
| 3. Adjusted BMI T4 | 0.75** | 0.86** | - | - | - |
| 4. BMI change T3-T2 | -0.30** | 0.26** | 0.18** | - | - |
| 5. BMI change T4-T3 | 0.03 | -0.01 | 0.49** | -0.08* | - |
| 6. BMI change T4-T2 | -0.20** | 0.18** | 0.50** | 0.67** | 0.69** |
| *Note. N* = 1306, ** p < 0.001, * p < 0.01. | | | | | |
